# Dr. W. H. Monagan

**Published:** 1918-12

**Authors:** 


**Dr. W. H. Monagan,** died of influenza Oct. at his home in Mount Alton, Pa.

